# Chemotactic Ligands that Activate G-Protein-Coupled Formylpeptide Receptors

**DOI:** 10.3390/ijms20143426

**Published:** 2019-07-12

**Authors:** Stacey A Krepel, Ji Ming Wang

**Affiliations:** Cancer and Inflammation Program, Center for Cancer Research, National Cancer Institute at Frederick, Frederick, MD 21702, USA

**Keywords:** formyl peptide receptors, ligands, diseases

## Abstract

Leukocyte infiltration is a hallmark of inflammatory responses. This process depends on the bacterial and host tissue-derived chemotactic factors interacting with G-protein-coupled seven-transmembrane receptors (GPCRs) expressed on the cell surface. Formylpeptide receptors (FPRs in human and Fprs in mice) belong to the family of chemoattractant GPCRs that are critical mediators of myeloid cell trafficking in microbial infection, inflammation, immune responses and cancer progression. Both murine Fprs and human FPRs participate in many patho-physiological processes due to their expression on a variety of cell types in addition to myeloid cells. FPR contribution to numerous pathologies is in part due to its capacity to interact with a plethora of structurally diverse chemotactic ligands. One of the murine Fpr members, Fpr2, and its endogenous agonist peptide, Cathelicidin-related antimicrobial peptide (CRAMP), control normal mouse colon epithelial growth, repair and protection against inflammation-associated tumorigenesis. Recent developments in FPR (Fpr) and ligand studies have greatly expanded the scope of these receptors and ligands in host homeostasis and disease conditions, therefore helping to establish these molecules as potential targets for therapeutic intervention.

## 1. Properties of FPRs

Formylpeptide receptors (FPR, Fpr for expression in mice) are G-protein-coupled receptors and were incidentally the first GPCRs to be identified in neutrophils [1]. Though initially cloned from neutrophils, FPRs have since been identified in macrophages, endothelial cells, intestinal epithelial cells, fibroblasts, and others [2,3,4,5]. Humans possess three different forms of FPRs: FPR1, FPR2, and FPR3. FPR1 was the first named of the receptors, and it was initially discovered as the receptor for the formylated bacterial product formyl-methionine-leucyl-phenylalanine (fMLF), the name of which gave rise to the naming of the receptor in question [6]. FPR1 is most highly expressed in cells in the bone marrow and immune system, though it has some expression notable in cells of the lungs, brain, and gastrointestinal (GI) tract, among others [7,8]. FPR2 was the second discovered of these receptors, but it tends to be the more ubiquitously expressed of the two. The expression is primarily in cells of the bone marrow, immune system, GI tract, female organ tissues, and endocrine glands, though there are also some lower levels of expression in cells of the brain, liver, gallbladder, and pancreas [8,9]. There is little known about the biological significance of FPR3, and very little research has been done to elucidate its role. This receptor is mainly expressed in monocytes and dendritic cells but not neutrophils, and it resides in intracellular vesicles rather than on the cell surface like its counterpart receptors. FPR3, contrary to the other FPR variants, also has only one known endogenous peptide agonist [10,11].

The primary roles of FPRs involve cell chemotaxis in response to agonists, and new research has shown that they even contribute to direct phagocytosis of bacteria by neutrophils [12,13]. Activation of such receptors is also important for wound healing and gut development [14,15]. However, while FPRs were initially thought to only be responsible for neutrophil chemotaxis, FPR1 and FPR2 have both been shown to play pivotal roles in the progression of multiple diseases. For example, FPR2 may promote the malignancy of colon cancer, while FPR1 has similarly been tied to the progression of glioblastoma [16,17]. Conversely, FPR1 has demonstrated tumor suppressor functions in gastric cancer [18]. With such dual roles in cancer progression, it is clear that further mechanistic studies would contribute greatly to our understanding of FPRs, thus potentially leading to new therapeutics. While aberrant expression or activation of FPRs can be detrimental, somewhat contradictory findings demonstrate that constitutively active FPR was indispensable in the defense against the formation of biofilms by *Candida albicans*, as well as aggressive infiltration by *Vibrio harveyi* [19]. FPR-mediated cell activation does not solely include chemotactic and pro-inflammatory responses to pathogens, as activation also plays a very important role in the protection against other pathologies. Gobbetti et al. [20] demonstrated that Fpr2 confers protection against sepsis-mediated damage in mice. Furthermore, FPRs have been reported to mediate anxiety-related disorders and the resultant altered behaviors [21]. Lastly, a novel discovery for FPRs suggests that they act as mechanoreceptors in arteries, making them critical for proper arterial plasticity [22]. Given such diverse functions of these receptors, it should come as no surprise that FPRs respond to a plethora of ligands with diverse classifications. 

While most FPR ligands induce cell chemotaxis, calcium flux, and even phagocytosis, they stimulate many other cell functions as well [23]. For instance, some ligands elicit inflammatory processes for the clearance of infection, recruitment of immune cells, etc. while other ligands activate pro-resolving, anti-inflammatory pathways. There are few chemoattractant GPCRs capable of transmitting both pro- and anti-inflammatory signals. This duality in FPR2 is initially determined by the nature of the ligands. Bacterial and mitochondrial formylated peptides are among those that classically activate a pro-inflammatory cell response to clear invaders and tissue damage, while Annexin A1 (Anx A1) and Lipoxin A4 (LXA_4_) are some of the better-known anti-inflammatory FPR2 ligands [24]. Cooray et al. [25] demonstrated that the switch between FPR2-mediated pro- and anti-inflammatory cell responses is due to conformational changes of the receptor upon ligand binding: binding of anti-inflammatory ligands such as Anx A1 caused FPRs to form homodimers, which led to the release of inflammation-resolving cytokines like IL-10. Conversely, inflammatory ligands such as serum-amyloid alpha (SAA) did not cause receptor homodimerization. 

Though some of the diverse FPR (Fpr) ligands are small-molecules or non-peptides, the majority are small peptides that are either synthetic or natural with origins ranging from host and multicellular organisms to viruses and bacteria. These peptides have been extensively studied and patterns of recognized elements have begun to emerge. As is demonstrated below, the presence of formylated methionine in the peptide is generally an activator of FPR1, while FPR2 is less dependent upon this particular residue [26]. Expanding upon this, Bufe et al. [27] concluded that FPR recognition of bacterial peptides requires either a formylated methionine at the N-terminus or an amidated methionine at the C-terminus of a peptide, though they believe that as a general principle, the secondary structure rather than the primary sequence is important for recognition of the highly diverse ligands by FPR. One class of ligands which shall be shortly discussed is comprised of phenol-soluble modulins, and examination of these has led to the conclusion that FPR1 favors short, flexible structures while FPR2 has binding preference for longer peptides which are amphipathic in nature and may contain alpha helices [28]. The class of ligands mentioned above represents a small proportion of the known FPR agonists today, and this review will focus on formylated, microbe-derived, and mitochondrial peptides, as well as host and non-microbial, non-host peptides. Host-derived non-peptides, as well as synthetic or small-molecule ligands will also be discussed.

## 2. Formylated Peptides

The prototypic FPR ligand is fMLF, which was the first classified FPR agonist and also represents the shortest sequence to elicit a potent receptor response. While Fpr2 is generally considered the more promiscuous FPR, fMLF preferentially activates FPR1 with high affinity [29]. It has been shown that non-formylated bacterial peptides are much less potent than their formylated counterparts; a suggested reason for this is due to the use of formyl-methionine as a Gram-negative start codon, therefore marking a protein as pathogenic from the perspective of the host immune system [30]. There are many derivatives of fMLF which elicit FPR responses, many of which preferentially activate FPR2 rather than FPR1. Such derivatives include the peptide sequences fMLFII, fMLFIK, fMLFK, and fMLFW, among others [31]. Liu et al. [32] summarized many other formylated peptides that elicit responses through both FPR1 and FPR2, including f-MIFL, f-MIVIL, f-MIGWII, and f-MFEDAVAWF. Dozens of similar peptides have been isolated from equally numerous bacterial genera including *Streptococcus*, *Haemophilus*, *Salmonella*, *Hydrogenobacter*, *Listeria*, *Neisseria*, *Staphylococcus*, and others [27]. PSMα is another formylated peptide that has shown great efficacy in FPR activation. Thus far, the phenol-soluble modulins that have demonstrated a capacity for FPR activation include β2, α1, and α2, all of which are virulence factors isolated from *Staphylococcus aureus*. All three peptides activate FPR2, though the structural basis for activation remains unknown [33]. 

It is interesting to note that mammalian cell mitochondria, well-known for being bacterial in origin, also contain peptides that elicit FPR-mediated responses. However, while bacterial formylated peptides are considered pathogen-associated molecular patterns (PAMPs), the mitochondrial peptides are generally associated with cellular damage and are thus considered damage-associated molecular patterns (DAMPs) that elicit an inflammatory response [34]. These peptides, some of which include MMYALF, MFADRW, and Nle-LF-Nle-YK, have been tied to constriction of airways in the lungs, as well as neutrophil accumulation and other proinflammatory responses [35,36]. Likewise, lung diseases—including acute respiratory distress syndrome—have been found to have a higher presence of formylated mitochondrial peptides in bronchoalveolar lavage fluid, suggesting that lung inflammation is tightly tied to Fpr1 activation via these DAMPs [37]. Mitocryptide-2 (MCT-2) is another mitochondrial peptide. It is related to Cytochrome B of the electron transport chain and activates FPR2. Interestingly, while the N-terminal formylated methionine is entirely necessary for FPR2 activation by MCT-2, the carboxy-terminal residues are also important for receptor activation. Additionally, sequence analysis has revealed that the presence of the residues Thr7 and Ser8 can activate FPR2, though the peptides were most potent with Ile7 and Asn8 residues [38]. 

Another important mitochondrial peptide is the nicotinamide adenine dinucleotide (NADH) reductase subunit 1 (ND-1), which elicits a strong inflammatory response via FPR1 [39]. Bufe and Zufall [40] have successfully created a model that accurately predicts known mitochondrial peptide agonists for FPRs. Therefore, there is a strong likelihood for the discovery of many new mitochondrial, host-derived FPR agonist peptides. Another DAMP that elicits FPR-mediated responses is Mitochondrial Transcription Factor A (Tfam). While this necrosis-associated peptide has been previously shown to activate FPRs, activation does not appear to play a crucial role in the inflammatory responses of monocytic microglia in the brain; this may be due to lower FPR expression in this cell type [41,42]. The formylated peptide ligands for FPRs (Fprs) are listed in Table 1, and the mitochondrial peptides are listed in Table 2. 

## 3. Microbe-Derived Peptides

Table 3 lists the microbe-derived FPR (Fprs) ligands. While formylated peptides first drew the attention of the scientific community to FPRs (Fprs), there are many other bacterial/viral peptides that are not necessarily formylated but which nevertheless elicit receptor responses. Although the majority of formylated microbial peptides preferentially activate FPR1, the preferred receptor for non-formylated peptides is FPR2 [26]. A large percentage of these non-formylated microbe-derived peptides are viral, and many of them are derived from the Human Immunodeficiency Virus (HIV) envelope proteins, including gp41 T20/DP178, gp41 T21/DP107, gp120 V3 loop, gp41 N36, gp120 F, and gp41 MAT-1 [44,45,46]. Despite the potential importance of FPRs in HIV research, very little work has been done to further explore this connection. However, Li et al. [47] demonstrated that persistent FPR activation desensitized host CCR5 and CXCR4 co-receptors to HIV proteins, thus reducing viral entry and subsequent replication. Still other viruses, including Hepatitis C Virus, HKU-1 coronavirus, and Herpes Simplex Virus, produce chemotactic ligands C5a, N-formyl HKU-1 coronavirus peptide, and gG-2p20, respectively, for FPR1 or FPR2 activation [48,49,50]. There is, however, some argument as to the efficacy of the Herpes Simplex viral peptide as an FPR agonist, as the overlapping sequence gG-2p19 was unable to definitively demonstrate that FPR activation played a significant role in the NK response to this virus [51]. 

Mills [49] used the sequence homology of T20/DP178 to further determine that the OC43 Coronavirus, 229E Coronavirus, NL36 Coronavirus, and even the Ebola Spike Protein were all peptides with aromatic-rich domains that elicited FPR-dependent cell activation. Interestingly, when examined from the context of the FPRs rather than the ligands, it was found that domain variability in the receptors determined ligand binding and subsequent cellular responses. This led to the conclusion that the variability of receptors among individuals might predispose or protect against certain viral infections, the susceptibility of which may be determined by receptor activation. In terms of non-viral and non-formylated microbe-derived peptides, there are few FPR agonists. Certain peptides from different strains of *Enterococcus faecium* have demonstrated FPR activation properties, though the ligand activity is not entirely predictable based on structure. Interestingly, *E. faecium* strains that are resistant to vancomycin contain potent FPR2 agonists, suggesting a potential role for FPR2 in antibiotic-resistant infections [52]. 

## 4. Host-Derived FPR Ligands

There are many different host-derived ligands that elicit strong FPR responses, though they have different biological implications. Misfolded proteins implicated in a variety of pathologies are one class of host-derived proteins eliciting FPR activation. Amyloid β-42 (Aβ-42), a peptide fragment well-documented in Alzheimer’s Disease (AD), interacts with FPR2. Additionally, part of FPR2’s role includes interaction with the Macrophage Receptor with Collagenous Structure (MARCO) scavenger receptor, which is responsible for reducing inflammation and alleviating inflammation-associated symptoms [60]. Interestingly, the symptoms that are viewed as a hallmark of AD are due to this same ligand binding an entirely different group of receptors which do not internalize them, therefore leading to an increased inflammatory pathology [61]. The prion peptide fragment, PrP_106-126_ also interacts with FPR2 on astrocytes and microglia, and the internalization of this peptide is detrimental to the host and contributes to disease progression [62]. Another neuropeptide that activates FPR2—though other studies claim it also activates FPR1—is the pituitary adenylate cyclase-activating polypeptide 27 (PACAP27), which has been shown to induce migration and Ca^2+^ mobilization, as well as upregulation of CD11b in neutrophils [63,64]. Another FPR agonist, the Vasoactive Intestinal Peptide (VIP), activates monocytes via FPR2 and may initiate an inflammation-resolving process [64,65].

Other host-derived FPR ligands are CKβ8-1 and the SHAAGtide sequence, as well as various uPAR domains from the Urokinase-Type 1 Plasminogen Activator Receptor (uPAR). CKβ8-1 is also known as the CCL23 chemokine, and it acts as an agonist for FPR2, along with its truncated N-terminal peptide called the SHAAGtide sequence that activates a chemokine GPCR [66,67]. It has been demonstrated that several uPAR peptides elicit FPR responses, including uPAR_88-92_, uPAR_84-95_, D2D3, and the SRSRYp sequence [68,69,70]. These sequences may foster the transition between fibroblasts to myofibroblasts, therefore increasing the pathology of fibrosis via FPR2 activation [68]. Another host-derived peptide, F2L, is derived from the N-terminus of the heme-binding protein, HEPB1. As the sole agonist specific for FPR3, F2L activates macrophages and possibly dendritic cells (DCs) as well [71]. However, other studies have demonstrated that FPR2 in neutrophils also exhibits a moderate affinity for F2L, though in this scenario F2L appears to have an inhibitory rather than a stimulatory effect [72,73]. A newer FPR ligand is Family with Sequence Similarity 3 (Member D), or FAM3D. This chemokine-like peptide is most highly expressed in the GI tract, though it is also expressed in cells of the immune system [8,74]. FAM3D has demonstrated a high affinity for both FPR1 and FPR2 and has been implicated in playing an important role in both inflammation and GI homeostasis via FPR activation [75]. Additional studies have found that FAM3D may also be involved in the beneficial role of glucagon secretion in Type 2 diabetes, as well as the detrimental development of abdominal aortic aneurysms [76,77].

In addition to endogenous peptides associated with cell-surface proteins and functional units, there are many ligands that are secreted by cells in response to tissue damage. Annexin-1 (AnxA1), also called Lipocortin-1, is an anti-inflammatory protein which is upregulated as a result of the stress responses of multiple host systems [78]. Some studies demonstrate AnxA1 to be an FPR1 agonist, while others show it as an FPR2 agonist; hence, it likely activates both. One study demonstrated its role in the attenuation of rheumatoid arthritis symptoms by decreasing fibroblast-like synoviocyte proliferation via FPR2 [79]. However, another study showed that AnxA1 initiated autocrine signaling in breast cancer via FPR1 and led to an increase in tumor growth and metastasis [80]. Additionally, the absence of AnxA1 has been tied to increased disease severity in both rheumatoid arthritis and obstructive pulmonary disease, leading to the hypothesis that treatment with exogenous AnxA1 may help reduce symptoms associated with different inflammatory pathologies [81,82]. In addition to AnxA1, multiple derivatives of the parent protein, including Ac_1-25_, Ac_2-26_, and Ac_9-25_, activate FPR2 [83,84,85]. These peptides have protective effects in ischemia-induced lung injury and atherosclerosis [84,86]. As with its pro-survival property, AnxA1 acts as a double-edged sword: secretion of either AnxA1 or Ac_2-26_ by tumor-associated fibroblasts induces the acquisition of stem-like features in prostate cancer cells, thus leading to a worse prognosis [87]. 

Serum-amyloid alpha (SAA) is an endogenous FPR2 agonist secreted by liver or macrophages in response to inflammatory stress and, more notably, tissue damage. In endothelial cells, SAA enhances the expression and activity of Tissue Factor—a protein necessary for clotting and wound repair—while additionally inhibiting the activity of Tissue Factor Pathway Inhibitor. Both functions were demonstrated to be the result of FPR2 activation [88]. SAA, via FPR2 activation, additionally increases the production of the wound-healing chemokine, CCL2, by vascular endothelial cells [89]. More recent studies have demonstrated the role of an SAA-FPR2 axis in neovascularization in the cornea as well [90,91]. 

Human LL-37 is an antimicrobial peptide that induces Cxcl13 and Tnfsf13b transcription, as well as B cell activation and proliferation via FPR2. It also contributes to the maintenance of B-cell germinal centers in Peyer’s Patches of the gut [92]. LL-37 also promotes the growth of both colorectal and ovarian cancer cells [93,94]. The murine homologue of LL-37, Cathelicidin-related antimicrobial peptide (CRAMP), is similarly an Fpr2 agonist and has been shown to promote atherosclerosis and DC maturation [95,96]. CRAMP also plays a pivotal role in maintaining the homeostasis of the colon mucosa and microbiota balance, demonstrating its potential as a therapeutic molecule [97]. The list of host-derived FPR (Fpr) ligands is shown in Table 4.

## 5. Synthetic Peptides and Non-Peptide Small Molecules

By far the most extensive category of FPR ligands includes the synthetic and small-molecule ligands, of which there are over 40 currently known, as shown in Table 5. W-peptides are among the better-known synthetic peptides acting as FPR agonists, and they include the sequences WKYMVm-NH_2_, WKYMVM-NH_2_, as well as many derivatives. Bufe et al. [27] demonstrated that both FPR1 and FPR2 mediate Ca^2+^ mobilization responses in leukocytes to more than 20 different combinations and derivatives of the W-peptide. A breakdown of the data suggests that certain residues in the peptide sequence are more important than others: C3 tyrosine, C4 methionine, and C6 D-methionine are all required for ligand activity, as is the carboxy-terminal NH_2_. Peptides may additionally be shortened on the N-terminus by two amino acids or elongated by three amino acids before FPR activation capacity is severely diminished. While the applications of WKYMV-sequences have been little explored, one recent study showed that the activation of FPR2 by WKYMVm may enhance the homing of endothelial cells, thus improving tissue healing, especially in ischemic neovasculogenesis in injured limbs [3]. Interestingly, another study showed that WKYMVm was capable of desensitizing HIV coreceptors CXCR4 and CCR5, therefore decreasing the entry of HIV-1 into macrophages and CD4+ T cells [47]. 

M-peptides are another subclass of synthetic/peptide library isolates and include MMK-1 and MMWLL. MMK-1, an FPR2 agonist, is by far the more commonly used peptide, and studies have shown that it may be useful as an anti-anxiety drug, as well as a drug to counteract hair loss from chemotherapy [98,104]. However, there has been some concern about its use in certain drug regimens, as it may amplify the response of monocytes to SIO_2_-coated nanoparticles, making it an important player in calculating the proper dosing when using such nanoparticles [105]. MMWLL is another M-peptide specific for FPR1. It is not classically a formylated peptide, though the addition of a formylated methionine induces a more potent FPR1 response than even the classic prototypic fMLF [106]. A much newer class of synthetic peptides are the FPR1-agonistic AApeptides, based on the general structure of N-acylated-N-aminoethyl amino acid residues. There are three different AApeptide subgroups called the α-peptides, α-AApeptides, and γ-AApeptide, all of which have different R-groups at the designated α or γ position. Most of these derivatives of AApeptides induce Ca^2+^ mobilization in rat basophil leukemia (RBL) cells transfected with human FPR1, though the γ-AApeptide Compound 7 at 10 μM elicited a more potent cell response than fMLF at the same concentration, making it a reasonably high-affinity ligand for FPR1 at this concentration, though not at lower concentrations [107]. See Table 5 for all synthetic peptides.

Some newer, non-peptide synthetic compounds being studied are various derivatives of ureidopropanamide molecules. They have demonstrated a capacity for protection against LPS-induced microglial death via an FPR2-dependent pathway, and pre-treatment with concentrations as low as 1 μM showed the protective effects [24]. Thus, these ligands show promising potential as treatment for diseases associated with inflammation in the Central Nervous System (CNS). Another pro-resolving synthetic ligand is the quinazolinone derivative Quin-C1. It is an Fpr2 agonist and has been shown to be effective at reducing inflammatory cytokines and clearing neutrophils and lymphocytes in murine models of lung injury [114]. Schepetkin et al. [115] demonstrated that three different synthetic molecules are agonists for both FPR1 and FPR2. Two of these are bombesin-related BB_1_/BB_2_ antagonists called PD168368 and PD176252, and they induce Ca^2+^ flux as well as neutrophil degranulation with EC50 values in the nanomolar range, thus making them potent FPR agonists. The third agonist is the Cholecystokinin-1 receptor agonist A-71623, which exhibits FPR1 and FPR2 agonism, though with a much higher EC50. In structural studies, these ligands were cross-reactive with FPR1/2 and possessed both Trp and N-phenylurea moieties. This led to the hypothesis that the combination of moieties greatly increases the chance that an agonist will activate both receptors.

Kirpotina et al. [116] screened over 6000 compounds and isolated nearly 30 different FPR1 and/or FPR2 agonists, all of which are derivatives of acetohydrazide, 2-(N-piperazinyl)acetamide, N’-phenylurea, and benzimidazole. The acetohydrazide derivatives (compounds AG-07/7, AG-09/92, AG-09/96, AG-09/101, and AG-09/102) and N-phenylurea derivatives (AG-09/3, AG-09/4, AG-09/73 through AG-09/77, and AG-09/82) are all FPR2-specific, though the acetohydrazide compounds tend to have lower efficacies on average. Also, the benzimidazole derivatives are either FPR1-specific (AG-09/1, AG-09/2, AG-09/13, AG-09/18, AG-09/19, and AG-09/21) or are agonists for both FPR1 and FPR2 (AG-09/16, AG-09/17, AG-09/20, and AG-09/22 through AG-09/24); no FPR2-specific benzimidazole derivatives have yet been identified. Pyridazines are another class of non-peptide, synthetic molecules which can have many different derivatives. Currently, only two compounds have been identified as potent mixed FPR1 and FPR2 agonists with an EC50 of around 2 μM each. These two compounds are referred to as compounds 8b and 8c with the Pyridazin-3(2H)-one structure. Additionally, they have R substitutions of SCH3 and OCH3, as well as R1 substitutions of I and SCH3, respectively. Both R and R1 substitutions are on substituted benzene rings [117]. (See Table 6 for the list of synthetic/small molecule non-peptide agonists).

## 6. Ligands from Non-Human Sources

As the search for disease treatments continues, many investigators have turned to developing compounds isolated from various plants and animals for potential therapeutic uses. Some of these new compounds have been shown to activate human or murine FPRs. The first of these is a series of compounds isolated from the centipede *Scolopendra subspinipes mutilans*, which has classically been used in Oriental medicine and is now being studied for therapeutic potential [126]. New studies show that compounds Scolopendrasin III and V both cause human neutrophil migration via FPR1, while Scolopendrasin IX seems to work through FPR2 to promote neutrophil chemotaxis. The two former compounds have not yet been studied for effectiveness against particular pathologies, though Compound IX has traditionally been an effective treatment for rheumatoid arthritis in Oriental medicine. New evidence confirms this activity, citing the activation of FPR2 as the mechanism [9,127]. Temporins are another class of FPR agonists and consist of antimicrobial peptides isolated from the *Rana Temoraria* frogs. Temporin A and Rana-6 are two such peptides, and both activate FPR2 to promotion leukocyte migration. There are also two distinct synthetic peptides, I4S10-C and I4G10-C, that are modeled after temporins and activate FPR2 [128].

Rubimetide is a peptide (Met-Arg-Trp) isolated from the digest of Rubisco in spinach. While it has been studied for some time, it has just recently been classified as an FPR2 agonist and has further demonstrated an ability to produce anxiolytic-like effects, thus alleviating some symptoms associated with anxiety [98]. The same investigators also isolated soymetide from the α’ subunit of β-conglycinin from soybeans and then demonstrated its activity as an FPR1 agonist [129]. Furthermore, the antimicrobial peptides Piscidin-1 and -3, which were isolated from fish, have been shown to induce myeloid cell chemotaxis via both FPR1 and FPR2. As a testament to the harms of water pollution by metals, this study also demonstrated that conjugation of Cu^2+^ with either of these compounds reduces the chemotactic activity of mammalian neutrophils [130] (See Table 7 for the list of peptides from other non-human sources).

## 7. Concluding Remarks

FPRs are a class of seven-transmembrane, G-protein-coupled receptors (GPCRs) that interact with a remarkably diverse range of ligands. As demonstrated, these ligands may originate from pathogens, the host, the synthetic peptide or compound library, or even non-host multicellular organisms. With such diverse agonist binding capacity, it is not surprising that FPRs may be either detrimental or beneficial in different pathophysiological conditions. Though the majority of these agonists have been known for more than a decade, newer studies are finding novel roles for these ligands in treatments for conditions ranging from anxiety and mental health disorders to arthritis and wounds. The field of FPR agonist studies has demonstrated the potential of these molecules to have therapeutic mechanisms useful for medicine. In addition to the vast number of agonists summarized here, there are also extensive lists of antagonistic ligands that may also provide protective mechanisms in various diseases [23,44]. Thus, further exploration of FPRs and ligands as therapeutic targets would be highly beneficial to diseases including cancer, sceptic shock, arthritis, and many other inflammatory pathologies.

## Figures and Tables

**Table 1:** Formylated bacterial peptide agonists for formylpeptide receptors (FPRs) (Fprs).

Agonist	Classification	Receptor	Citation
MLF		FPR1	[29]
MIFL		FPR1, FPR2	[32]
MIVIL		FPR1, FPR2	
MIGWI		FPR1, FPR2	
MIVTLF		FPR1, FPR2	
MIGWII		FPR1, FPR2	
MFEDAVAWF		FPR1, FPR2	
MLFK (-II, -IK, -K, -W)		FPR2	[31]
*Streptococcus* f-MGFFIS		FPR1, FPR2	[27]
*bacillus* f-MKNFKG		FPR 2	
*Salmonella* f-MAMKKL		FPR 1	
*Haemophilus* f-MVMKFK		FPR1, FPR2	
*Psychrosomonas* f-MLFYFS		FPR1, FPR2	
*Desulotomaculum* f-MLFYLA		FPR1, FPR2	
*Borrelia* f-MLKKVY		FPR1, FPR2	
*Vibrio* F-MVKIIF		FPR1, FPR2	
*Clostridium* f-MKKNLV		FPR 2	
*Streptomyces* f-MVPISI		FPR1, FPR2	
*Hydrogenobacter* MKKFLL		FPR 2	
*Listeria* MKKIML		FPR1, FPR2	
*Desulfovibrio* MKFCTA		FPR1, FPR2	
*Neisseria* MKTSIR		FPR1, FPR2	
*Staphylococcus* MFIYYCK		FPR 1	
f-NLPNTL		FPR 1	[43]
PSMα peptides β2, α1, and α2	Staphylococcus aureus	FPR 2	[33]

**Table 2:** Mitochondrial peptide ligands for FPRs (Fprs).

Agonist	Classification	Receptor	Citation
MMYALF	Formylated	FPR 2	[35]
MLKIV	formylated	FPR 2	
MYFINILTL	Formylated	FPR 2	
MFADRW	Formylated	FPR 2	
Nle-LF-Nle-YK		FPR 2	
Mitocryptide-2 [MCT-2]	Cytochrome B	FPR 2	[38]
ND1	NADH reductase subunit 1	FPR1	[39]
TFam	Mitochondrial Transcription factor	FPR1	[41]

**Table 3:** Microbe-derived peptide ligands for FPRs (Fprs).

Agonist	Classification	Receptor	Citation
Hp2-20	Helicobacter	FPR2	[53]
T20/DP178	HIV gp41	FPR1	[45]
T21/DP107	HIV gp41	FPR1, FPR2	[54]
V3 peptide	HIV Gp 120	FPR2	[55]
N36 peptide	HIV Gp 41	FPR2	[56]
F peptide	HIV Gp 120	FPR2	[57]
MAT-1	HIV gp41	FPR2	[46]
gG-2p20	Herpes Simplex Virus	FPR1	[50]
N-formyl HKU-1 coronavirus peptide	HKU-1 virus	FPR1	[49]
*Listeria* peptides		FPR1	[26]
*Enterococcus faecium* proteins		FPR2	[52]
PrP_106-126_		FPR2	[58]
C5a HCV peptide	Hep C Virus	FPR2	[48]
OC43 coronavirus protein	OC43 Coronovirus	Unknown	[49]
229E Coronavirus protein	229E Coronavirus	Unknown	
NL36 Coronavirus protein	NL36 Coronavirus	Unknown	
Spike Protein	Ebola virus	Unknown	
Influenza A Virus	Annexin A1 surface protein	FPR2	[59]

**Table 4:** Host-derived FPR (Fpr) ligands and their classification.

Agonist	Classification	Receptor	Citation
CKβ 8-1 [human CCL23]	CCL23 chemokine	FPR2	[66]
SHAAGtide	CKβ 8-1	FPR2	
Humanin		FPR2	[98]
F2L	Heme binding protein	FPR2, FPR3	[71,72,73]
SAA		FPR2	[90]
Annexin 1/ Lipocortin 1		FPR2	[78]
Ac_1-25_	Annexin 1	FPR2	[83]
Ac_2-26_	Annexin 1	FPR2	[84]
Ac_9-25_	Annexin 1	FPR2	[85]
Antiflammin-2 (AF-2)		FPR2	[99]
Aβ-42	Amyloid peptide	FPR2	[60]
D2D3	Urokinase receptor	FPR2	[70]
SRSRYp	Urokinase receptor	FPR2	
LL-37	Antimicrobial peptide	FPR2	[92]
PrP_106–126_	Prion protein	FPR2	[62]
PACAP27	Pituitary adenylate cyclase	FPR2, FPR1	[63,64]
uPAR_88-92_	Urokinase receptor	FPR2	[68]
uPAR_84-95_	Urokinase receptor	FPR2, FPR3	[69]
Cathepsin G		FPR1	[100]
FAM3D	Chemokine-like	FPR1, FPR2	[75]
FAM19A4	Chemokine-like	FPR1	[13]
ATLXA4 acetylsalicyclic acid-triggered	Arachidonic acid derivative	FPR2	[101]
Resolvin D1 (RvD1) and aspirin-triggered RvD1	Specialized pro-resolving lipid mediator	FPR2	[102]
Vasoactive intestinal protein		FPR2	[65]
Lipoxin-A4/Apsirin-triggered lipoxins	Fatty Acid	FPR2	[103]

**Table 5:** Synthetic peptide ligands for FPRs (Fprs).

Agonist	Classification	Receptor	Citation
AApeptidesα-peptides, α-AApeptides, and γ-AApeptide	N-acylated-N-aminoethyl amino acid residue	FPR1	[107]
CGEN-855A	TIPMFVPESTSKLQKFTSWFM-amide	FPR2	[108]
P2Y2Pal_IC2_ pepducin	P2Y_2_R pepducin	FPR2	[109]
F2Pal_10_	FPR2 pepducin	FPR2	[110]
GMMWAI	Gly-Met-Met-Trp-Ala-Ile-CONH_2_	FPR1	[111]
Lau-[[S]-Aoc]-[Lys-Bnphe]_6_-NH2	Lipidated peptomimetic peptide	FPR2	[112]
L-37pA	ApoA-I mimetic peptide	FPR2	[113]
MMHWFM	Met-Met-His-Trp-Phe-Met-CONH_2_	FPR1	[111]
MMHWAM	Met-Met-His-Trp-Ala-Met-CONH_2_	FPR2	[111]
MMK-1	Peptide library	FPR2	[98]
MMWLL	Peptide library	FPR1	[106]
WKYMVm(-NH2)	Peptide library	FPR1, FPR2	[27]
WKYMVM	Peptide library	FPR1, FPR2	
F2Pal_16_	FPR2 Pepducin	FPR2	[110]

**Table 6:** Small/molecule compounds functioning as FPR (Fpr) agonists.

Agonist	Classification	Receptor	Citation
Quin-C1	Quinazolinone derivative	FPR2	[114]
N-substituted benzimidazole 11		FPR2	[118]
Compound 8a	Pyridazin-3(2H)-one derivative 1	FPR1, FPR2	[117]
Compound 8b	Pyridazin-3(2H)-one derivative 2	FPR1, FPR2	
Chiral pyridazines	6-methyl-2,4-disubstituted pyridazine-3(2H)-ones	FPR1 [weak], FPR2	[119]
PD176252	Bombesin-related BB_1_/BB_2_ antagonist	FPR1, FPR2	[115]
PD168368	Bombesin-related BB_1_/BB_2_ antagonist	FPR1, FPR2	
A-71623	Cholescystokinin-1 receptor agonist	FPR1, FPR2	
1753-103	Synthetic library	FPR1	[120]
Compound (S)-17	Ureidopropanamide derivative (S)-3-(4-cyanophenyl)-N-((1-(3-chloro4-fluorophenyl]cyclopropyl]methyl)-2-(3-(4-fluorophenyl)ureido)propaniamide ((S)-17)	FPR2	[24]
1754-49	Synthetic library	FPR2	[120]
(S)-9a	3-(1H-Indol-3-yl)-2-(3-(4-nitrophenyl)ureido)propenamide derivative	FPR2	[121]
IDR1	KSRIVPAIPVSLL-NH2	FPR1	[122]
IDR-1002	VQRWLIVWRIRK-NH2	FPR2-uncertain	[123]
AG-09/1, AG-09/2, AG-09/13, AG-09/18, AG-09/19, and AG-09/21	Benzimidazole derivatives	FPR1	[116]
AG-09/16, AG-09/17, AG-09/20, and AG-09/22 through AG-09/24	Benzimidazole derivatives	FPR1, FPR2	
AG-09/3, AG-09/4, AG-09/73 through AG-09/77, and AG-09/82	N-phenylurea derivatives	FPR2	
AG-09/7, AG-09/92, AG-09/92, AG-09/96, AG-09/101, and AG-09/102	Acetohydrazide derivatives	FPR2 [low efficacy]	
Compound17a/17b/14a	Synthetic library	FPR1, FPR2	[124,125]
Compound43	Synthetic library	FPR1, FPR2	[79]

**Table 7:** Non-human, non-microbe-derived FPR (Fpr) ligands.

Agonist	Classification	Receptor	Citation
Scholopendrasin IX	Centipede Peptides	FPR2	[131]
Scholopendrasin III and V		FPR1	[127]
Rubimetide	Spinach	FPR2	[98]
Piscidin 1 and 3	From fish	FPR2	[130]
Temporin A	From frogs	FPR2	[128]
Rana-6		FPR2	
